# Real-world bisphenol A exposure not linked to microbiota dynamics in childhood obesity

**DOI:** 10.1128/msystems.00556-24

**Published:** 2024-07-03

**Authors:** Robyn L. Prueitt, Julie E. Goodman

**Affiliations:** 1 Gradient, Seattle, Washington, USA; 2 Gradient, Boston, Massachusetts, USA; University of California, San Diego, La Jolla, California, USA

**Keywords:** bisphenol A, gut microbiome, childhood obesity

## LETTER

Lopez-Moreno et al. (1) exposed fecal samples from a small number of normal-weight, overweight, and obese children to bisphenol A (BPA) and identified what they considered to be “BPA-tolerant” gut microbiota in all three study groups. The authors also reported that normal-weight children had more diverse microbiota, including bacteria with a high potential for enzymatic degradation of BPA, compared to the obese group. The authors suggested that BPA exposure could contribute to differences in the establishment of microbial communities in children with obesity compared to those with normal weight.

While the authors acknowledge that their findings are speculative and that more research on the topic is needed, we write this letter to clarify that this study does not provide any information regarding whether typical human exposures to BPA can affect the gut microbiome in a manner that is connected to obesity, nor does it show that children with obesity have a decreased ability to metabolize and excrete BPA from the body. The BPA exposure levels used in this study (20 and 50 ppm) are orders of magnitude higher than those typically encountered in humans through the ingestion of BPA. This is based on an estimated median intake of 27.2 ng/kg-day BPA in children, as derived from urinary BPA concentrations (2), which include all exposure sources of BPA. This intake is equivalent to 0.00272 mg over 3 days (assuming a child body weight of 33.4 kg). Fifty parts per million in 5 mL of bacterial media is equivalent to 250 mg BPA or almost 100,000-fold higher.

It is unlikely that *in vitro* exposure of fecal material to BPA concentrations that are five orders of magnitude higher than typical human exposures can recapitulate the conditions in the human gut *in vivo* and result in the same bacterial responses. In addition, there is no evidence to indicate that children or adults who are obese cannot metabolize BPA to the same extent as non-obese individuals. BPA has a low potential to bioaccumulate in the body, as oral exposure to BPA results in rapid and complete (~99%) metabolism and urinary excretion, with a half-life of less than 6 hours (3–6). Biomonitoring studies indicate similar or only slightly higher urinary levels of BPA in obese populations compared to non-obese populations (7). This may indicate higher food consumption and, thus, higher exposure in obese individuals, and there is no evidence that suggests impairment of BPA metabolism in obese populations compared to non-obese populations.

The study by Lopez-Moreno et al. (1) provides interesting data on the diversity of gut microbiota in children, but we think it is important to put these results into a real-world context. Given the *in vitro* design and extremely high BPA concentrations that are at least five orders of magnitude higher than typical human exposures, this study was hypothesis-generating and not necessarily (nor likely) indicative of conditions in the human gut with typical, everyday exposure to BPA from foods and other sources.
